# Predicting the risk of osteoporosis in older Vietnamese women using machine learning approaches

**DOI:** 10.1038/s41598-022-24181-x

**Published:** 2022-11-23

**Authors:** Hanh My Bui, Minh Hoang Ha, Hoang Giang Pham, Thang Phuoc Dao, Thuy-Trang Thi Nguyen, Minh Loi Nguyen, Ngan Thi Vuong, Xuyen Hong Thi Hoang, Loc Tien Do, Thanh Xuan Dao, Cuong Quang Le

**Affiliations:** 1 Department of Tuberculosis and Lung Disease, Hanoi Medical University, Hanoi, Vietnam; 2 Department of Functional Exploration, Hanoi Medical University Hospital, Hanoi, Vietnam; 3 ORLab, Faculty of Computer Science, Phenikaa University, Hanoi, Vietnam; 4 Department of Scientific Research and International Cooperation, Hanoi Medical University, Hanoi, Vietnam; 5 Administration of Science Technology and Training, Ministry of Health Vietnam, Hanoi, Vietnam; 6 Center for Development of Curriculum and Human Resources in Health Hanoi Medical University, Hanoi, Vietnam; 7 Hanoi Medical University Hospital, Hanoi, Vietnam; 8 Department of Orthopaedic, Hanoi Medical University, Hanoi, Vietnam; 9 Department of Neurology, Hanoi Medical University, Hanoi, Vietnam

**Keywords:** Predictive medicine, Metabolic bone disease

## Abstract

Osteoporosis contributes significantly to health and economic burdens worldwide. However, the development of osteoporosis-related prediction tools has been limited for lower-middle-income countries, especially Vietnam. This study aims to develop prediction models for the Vietnamese population as well as evaluate the existing tools to forecast the risk of osteoporosis and evaluate the contribution of covariates that previous studies have determined to be risk factors for osteoporosis. The prediction models were developed to predict the risk of osteoporosis using machine learning algorithms. The performance of the included prediction models was evaluated based on two scenarios; in the first one, the original test parameters were directly modeled, and in the second the original test parameters were transformed into binary covariates. The area under the receiver operating characteristic curve, the Brier score, precision, recall and F1-score were calculated to evaluate the models’ performance in both scenarios. The contribution of the covariates was estimated using the Permutation Feature Importance estimation. Four models, namely, Logistic Regression, Support Vector Machine, Random Forest and Neural Network, were developed through two scenarios. During the validation phase, these four models performed competitively against the reference models, with the areas under the curve above 0.81. Age, height and weight contributed the most to the risk of osteoporosis, while the correlation of the other covariates with the outcome was minor. Machine learning algorithms have a proven advantage in predicting the risk of osteoporosis among Vietnamese women over 50 years old. Additional research is required to more deeply evaluate the performance of the models on other high-risk populations.

## Introduction

Fragility fractures and their consequences are the most common signs of osteoporosis, the most prevalent disease related to the adult skeleton. Identifying patients at high risk of fracture prior to a fracture occurring is a critical component of osteoporosis care. This problem continues to be a major concern for researchers and physicians worldwide. Despite the fact that several algorithms have been created to either identify persons with osteoporosis or forecast their risk of fracture, concerns remain about their accuracy and usefulness. Scientific breakthroughs, such as machine learning technologies, are rapidly gaining acceptance as alternative approaches for improving risk assessment and existing practice.

Osteoporosis is a severe condition that primarily affects postmenopausal women. The standard-of-care test for osteoporosis includes estimating bone mineral density (BMD) in the proximal femur, the lumbar spine, and, in certain cases, the forearm using dual-energy X-ray absorptiometry (DXA). The BMD is then compared to that of a reference group, including a sex-matched and ethnicity-matched healthy, premenopausal adult population (e.g., how much lower it is regarding standard deviations, or the T-score) for diagnosis^1,2^. Professional organizations, such as the International Society for Clinical Densitometry, the United States Preventive Services Task Force, and the International Osteoporosis Foundation, all promote screening strategies for older women, but determining when and how to conduct the screenings is more contentious.

The Osteoporosis Self-Assessment Tool (OST) is one of the oldest and simplest ways to identify people at risk of osteoporosis. This tool uses *weight* and *age* to identify men and women in various populations likely to have osteoporosis^3–13^. For the Asian female population, prediction tools were developed by integrating the magnitude of the correlation between *age* and *weight* with BMD to estimate the likelihood of osteoporosis^5,14,15^.

By extending the number of factors used to determine osteoporosis, 12 input parameters, such as demography, lifestyle, and medical history were included in the Fracture Risk Assessment Tool (FRAX)^16^. Similarly, other complex tools, including ORAI^17^, SCORE^18^, ORISIS^19^, ABONE^20^ and MOST^21^, have incorporated additional characteristics in order to improve the performance of OST detection. Although combining numerous recognized risk factors was expected to improve the utility of screening tools, studies have revealed that basic tools, such as OST, might work as well as those with more complicated algorithms, while recent systematic reviews have emphasized the potential and limitations of these approaches.

### Machine learning models to predict risk of osteoporosis

Scientists worldwide recognize osteoporosis as a significant public health issue. While therapy can reduce fracture risk by 33% to 50%^22^, only a small percentage of patients, including those who have previously experienced osteoporotic fractures, receive the proper diagnosis and treatment. Furthermore, accurately identifying high-risk and/or high-cost patients in a fast and accurate way is expected to enhance effective healthcare management, as well as enhance clinical decision-making and to improve service planning and policy^23,24^. In recent decades, machine learning models have been increasingly integrated into osteoporosis prediction, along with the effective uses of healthcare big data, leading to improvements in the quality and efficiency of healthcare planning and delivery. Additionally, early illness identification (through simpler intervention and treatment, for example), customized health management, and the efficient detection of fraudulent behavior in healthcare are some of potential advantages^25^. Artificial intelligence (AI) technology has been developed based on mathematical modelling over years. AI software has been applied to different majors, including epidemiological survey^26^, drug discovery^27^, and diagnostic radiology^28^. At this point, the computer-assisted devices have been integrated to clinical routine practice to detect the abnormalities relating to respiratory diseases on chest X-ray images^29–31^. The on-site implementation of AI softwares proves its advantages to minimize the diagnostic bias, overcome the burnout issues and enhance the active case finding in community. Codlin et al. included 12 AI softwares to predict tuberculosis on chest X-ray images into the independent performance evaluation, which stated that a half of the AI softwares had the higher specificity values than ones of an intermediate radiologist^28^.

In a study by Erjiang et al.^32^, seven machine learning models—CatBoost, eXtreme Gradient Boosting, Neural Networks (NN), Bagged Flexible Discriminant Analysis, Random Forest (RF), Logistic Regression (LoR) and Support Vector Machines (SVM)—were implemented to derive the best fit models to differentiate between patients with and without osteoporosis using DXA T-scores. Ho-Pham et al. applied four machine learning models—artificial neural networks (ANN), LoR, SVMs and k-nearest neighborhood—to the BMD hip data of Australian women to identify hip fractures^33^. Ou Yang et al. implemented five ML models—ANN, SVM, RF, K-nearest neighbors (KNN), LoR—with many features, which were categorized into different areas related to bone health^34^. This study examined 16 input features for men and 19 input features for women in order to identify the relationship between the presence of certain features and risk of osteoporosis in a Taiwanese population. Other machine learning methods using OST to predict osteoporosis were reviewed by Ferizi et al.^1^.

Osteoporosis and the resulting fragility fractures are recognized as major public health issues throughout many developing countries, especially Vietnam. The lack of DXA equipment to diagnose osteoporosis in these countries requires a prediction model for individualized assessment. Ho-Pham et al. proposed a prediction model for individualized assessments of osteoporosis based on *age* and *weight* for men and women^14^. In this study, an LoR model using data from a population in Ho Chi Minh City was applied to develop the tool for each gender, with good accuracy. The researchers developed and validated a prediction model based on *age* and *weight* to estimate the absolute risk of osteoporosis in the Vietnamese population^14^. However, few studies have focused on OST-based prediction of osteoporosis in other areas in Vietnam. Our study’s primary objective was to build tools to assess the risk of osteoporosis from OST data in women over 50 years old in the Northern Vietnam. On the other hand, little is known about the performance of the model proposed by Ho-Pham et al.^14^ when applied on a new population. The Ho-Pham’s model proved its good accuracy during the internal validation^14^ whereas its performance to predict osteoporosis during an external validation was not noticeably reported. Therefore, our secondary objective was to independently validate the Osteoporosis Self-assessment Tool for Asians (OSTA) model and the model developed by Ho-Pham et al.^14^ on a new population. In addition to *age* and *weight*, our data now include *height, geographic location (urban/rural area) and blood test results of uric acid, cholesterol, creatinine, FT4, glucose, HbA1c, Ure, AST, TSH, calcium and GGT*. In their study, Ou Yang et al. concluded that specific blood test parameter is relevant to the OST (e.g., creatinine) to predict osteoporosis, while others (e.g., TSH) were of insignificant value in predicting osteoporosis in the Taiwanese population^34^. However, in contrast to Ou Yang et al. regarding the influence of TSH in North American patients, Jamal et al. recommended that patients with suspected osteoporosis based on their OST score undergoing the TSH test^34,35^. Therefore, the third objective of our research was to validate the conclusion of Ou Yang^34^ and Jamal^35^ using the dataset collected at Hanoi Medical University Hospital as well as to discover more novel factors linked to OST results.

### Significance of the study

The findings of this study would provide valuable evidence to strengthen the potentials of machine learning algorithms for use as decision making support tools in the context of the widely osteoporosis screening. The study would also present the supportive findings to promote the digital transformation of medical diagnosis in Vietnam. Additionally, the covariates which showed the significant contribution to the risk of osteoporosis would be pointed out and might be a valuable consideration for governmental policy makers in Vietnam.

## Methods

### Data source and participants

The data were retrospectively collected from the database of the health information system of the Hanoi Medical University Hospital from July 2018 to February 2021. The eligible population included women aged 50 years and older who underwent BMD testing at the study site and did not have metabolic bone disease (i.e., hypothyroidism, hyperthyroidism, Paget’s disease of bone, osteomalacia, renal osteodystrophy, osteogenesis), cancer with evidence of bone metastasis, chronic kidney failure requiring hemodialysis, history of a salpingectomy, bilateral femur surgery or were taking supplemental phosphonate, fluoride or calcitonin. We included in the prediction model the age, weight, height, blood test results and geographical factors of each patient. All of the indicators were collected by the time the participants had been registered by the doctors.

### Measurement

#### Physical index

Anthropometric measurements, including weight (kilograms) and height (meters), were measured following the Anthropometric Indicators Measurement Guide^36^. After participants removed their shoes, the Tanita WB-380H digital scale was used to measure their weight and height (they had been requested previously to wear light clothing). The measurements were repeated twice and then averaged.

#### Bone mineral density

We recorded the BMD values for all participants at different anatomical sites, including lumbar vertebrae (L1–L4) and the left and right femoral neck. The measurement was conducted using a Discovery Ci DXA system (Hologic, USA, 2019) using the parameters for the Japanese female population as a reference for our Asian population^37,38^. We converted the BMD values into T-scores, which we then transformed into categorized outcomes following the guidance of the World Health Organization^39^. Specifically, the outcomes were defined as follows: (1) T-scores of ≥  − 1.0 indicated normal BMD, (2) T-scores between − 1.0 and − 2.5 indicated osteopenia, and (3) T-scores of ≤  − 2.5 indicated osteoporosis^39^.

### Model development

We built four ML models based on LoR, SVM, RF, and NN. Each model was trained using two scenarios: (1) the original test indices and (2) the outliers of the test indices. In the first scenario, the models used the patient’s test parameters directly, whereas in the second scenario the patient’s test parameters were considered normal or abnormal prior to inclusion in the model. The thresholds for detecting the normal features were listed in Table 1.

**Table 1:** Thresholds for detecting normal features.

Blood test	Normal values
Acid uric (mmol/L)	202.3–416.5
Cholesterol (mmol/L)	< 5.2
Creatinine (mmol/L)	62–106
FT4 (pmol/L)	11.5–22.7
Glucose (mmol/L)	4.11–5.89
HbA1c (%)	4.8–5.9
Ure (mmol/L)	2.76–8.07
AST (U/L)	< 40
TSH (mU/L)	0.55–4.78
Calcium (mmol/L)	2.2–2.55
GGT (U/L)	8–61

All 4 models were built using scikit-learn software in Python 3.7. The LoR model was created to compare with the model by Ho-Pham et al., with the addition of the new features mentioned above. During each training section of the LoR, SVM, RF, NN models, the validation dataset was randomly split out from 20% of the training set to evaluate the performance of the models. The training was stopped after 100 epochs. The model that provided the best AUROC was applied for further analysis.

For hyperparameter tuning, Bayes search (with k-fold cross validation) was done for all models. For the LoR model, the solver type (Liblinear, newton-cg) and the regularization parameter C (from 1e−6 to 100 with log-uniform) were examined. For the SVM model, the kernel type (polynomial, radial basis function, sigmoid or linear), the regularization parameter C (from 1e−6 to 100 with log-uniform), and degree (from 1 to 4) for the polynomial kernel function were examined. For the RF model, the number of trees (from 100 to 500), and the maximum depth of the tree (from 3 to 11) were considered. For the NN model, the number of hidden layers (from 5 to 20), the solver (Adam, stochastic gradient descent), the maximum number of iterations (from 500 to 100), and the parameter alpha (from 1e−6 to 100 with log-uniform) were examined. All models were constructed with a balanced class weight. For each set of hyperparameters, fivefold split was repeated several times. We adopted the hyperparameter set that yielded the best result on the hold-out data. The hyperparameters of four models were listed in Table 2.

**Table 2:** The hyperparameters of four models.

Methods	First scenario	Second scenario
**LoR**		
Solver	Liblinear	Liblinear
C	0.001	0.014
Penalty	l2	l2
Best_score_	0.788	0.790
**SVM**		
Kernel	Poly	rbf
Degree	1	
C	100	4.196
Gamma	Scale	Scale
Best_score_	0.786	0.789
**RF**		
n_estimators	500	100
Max_depth	5	6
Best_score_	0.787	0.789
**NN**		
Solver	Adam	Adam
Alpha	98.130	2.433
Hidden_layer_sizes	11	12
Max_iter	574	746
Best_score_	0.786	0.789

### Data analysis

The area under the receiver operating characteristic curve (AUROC) was calculated for the test sets in each training process using the BMD as a reference, and the mean values of the AUROC were compared. The Brier score, precision, recall and F1-score were additionally calculated to evaluate the performance to predict osteoporosis of included prediction models. To compare the performance of our machine learning models and the OSTA tools, the OSTA score was calculated as $0.2 [Weight \left({\text{kg}}\right)-Age \left({\text{year}}\right)]$ on the test set. Then, the labels were generated by comparing the score to − 4 to classify patients as high risk. In addition, each model was processed 20 times with different random seeds. The metrics were calculated and averaged over all independent runs. In the LoR model, we determined the probability of osteoporosis according to the equation for women published by Ho-Pham et al.^14^. The labels on test set were created by comparing these number to 0.195. After that, the AUROC was also calculated and compared. The significance of all features was estimated by the Permutation Feature Importance of sklearn and then plotted using Python 3.7. In the Figs. 4, 5, 6, 7, 8, 9, 10 and 11, the horizon axis represented the decreases in the contribution to the osteoporosis prediction of each feature in the dataset. The larger the number was, the more the factor reduced the noise affecting the results, and therefore the more it contributed to whether the sample was properly classified. In addition to the Permutation Feature Importance, we used a chi-squared test to calculate the relationship among random features. This measurement can eliminate attributes that are more likely to be label-independent (osteoporosis or no osteoporosis) and were therefore irrelevant to the classification. The *p*-value in each table was calculated based on the chi-squared test. We chose the threshold of 0.05 for a type I error rate. The features with a *p*-value of ≥ 0.05 were independent of the labels, and they did not significantly contribute to the outcome of the prediction. Those with *p*-values of < 0.05 indicated a significant relationship to the prediction results. A *p*-value of < 0.01 indicated important features that directly affected the model’s classification.

### Ethical considerations

All the procedures conducted in this study aligned with the Ethical Review Board of the Hanoi Medical University (IRB approval No. HMUIRB563; Date: October 22, 2021). The study was approved by the Ethical Review Board of the Hanoi Medical University with the protocol number and the date of the IRB approval as mentioned above. The waiver of the informed consent was provided by the Hanoi Medical University ethics committee. All of the participants’ study data were masked. All the methods were carried out strictly following relevant guidelines and regulations.

## Results

**Figure 1:**
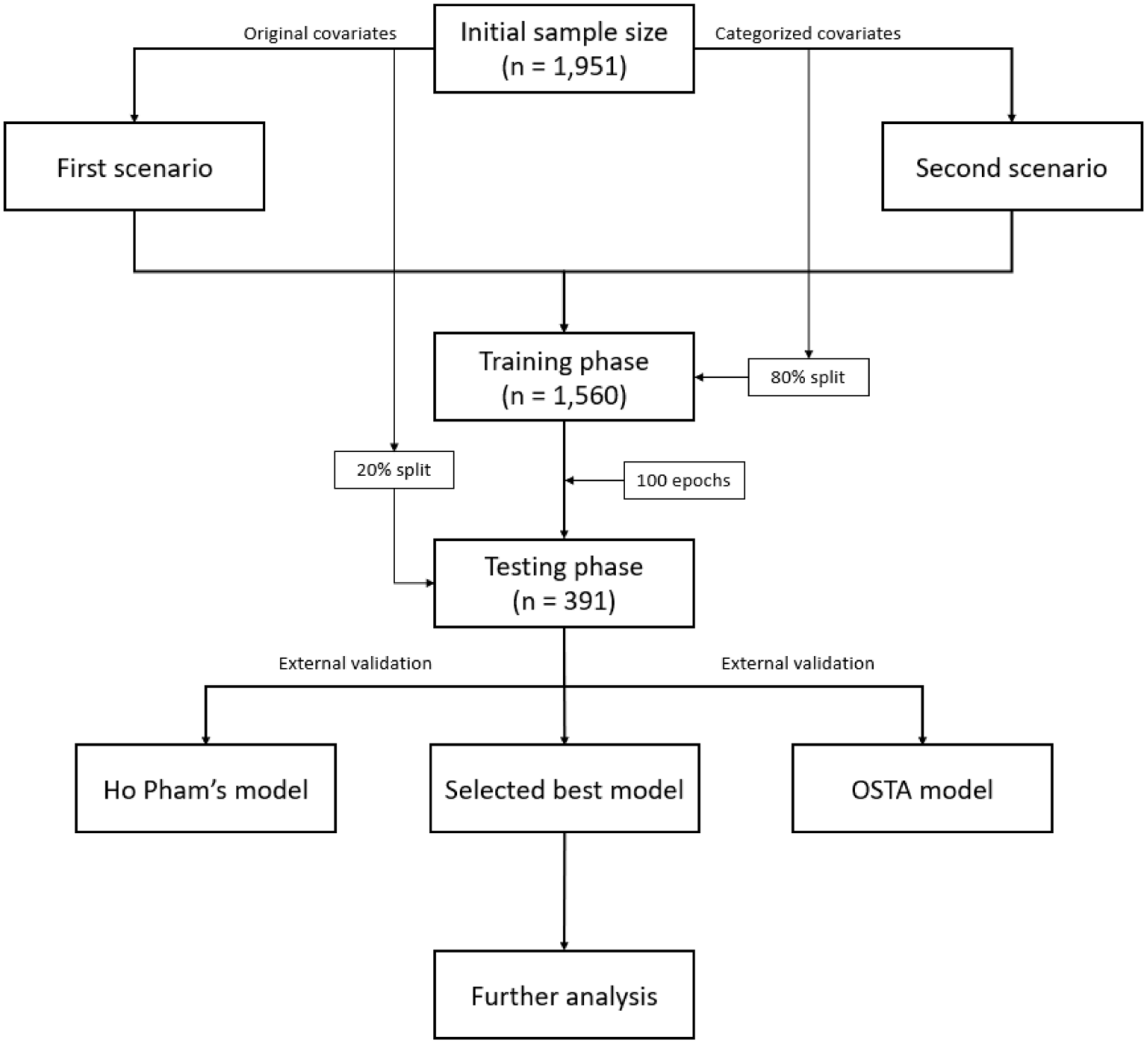
The diagram of the study procedure.

Figure 1 presents the procedure of sample collection, model development of the study. The initial dataset included 1951 participants. The training dataset was created from 80% of initial dataset and the testing dataset was split out from the remaining 20% of the initial data.

Tables 3 and 4 present the statistical distribution of the dataset. The models were trained on a dataset of 1560 patients (80% of the initial data) and tested on a dataset of 391 patients (20% of the initial data). In the blood test data of 1,951 female patients over 50 years of age, the percentage of patients with abnormal levels of uric acid, FT4, AST, urea, calcium and GGT were quite low at 4.87%, 2.31%, 3.95%, 3.48%, 2.82% and 1.33%, respectively. However, the corresponding numbers of cholesterol, creatinine, glucose, HbA1c and TSH were quite high, at 23.12%, 33.67%, 19.84%, 10.71% and 19.43%, respectively. The proportion of patients in urban and rural areas was similar, at 49.72% and 50.28%, respectively. The proportion of patients with diagnosed osteoporosis in the dataset was 28.91%.

**Table 3:** The statistical distribution of the data in the second scenario.

Blood test	Normal bone density (n = 1378, 71.092%) Normal–abnormal (%)	Decreased bone density (n = 564, 28.908%) Normal–abnormal (%)
Acid uric	95.674–4.326	93.794–6.206
Cholesterol	76.640–23.360	77.482–22.518
Creatinine	67.484–32.516	63.475–36.525
FT4	97.477–2.523	98.227–1.773
Glucose	81.471–18.529	76.950–23.050
HbA1c	89.690–10.310	88.298–11.702
Ure	97.621–2.379	93.794–6.206
AST	96.395–3.605	95.213–4.787
TSH	97.621–2.379	96.277–3.723
Calcium	97.909–2.091	95.390–4.610
GGT	98.414–1.586	99.291–0.709
Location	51.622–48.378	48.404–51.596

**Table 4:** The statistical distribution of the data in the first scenario.

Blood test	Normal bone density (n = 1378, 71.092%) Mean (standard deviation)	Decreased bone density (n = 564, 28.908%) Mean (standard deviation)
Age (years)	60.863 (7.373)	70.420 (8.393)
Weight (kg)	53.998 (7.018)	48.666 (7.665)
Height (cm)	152.047 (5.381)	147.753 (6.107)
Acid uric (mmol/L)	110.976 (155.049)	105.158 (157.538)
Cholesterol (mmol/L)	2.441 (2.787)	2.273 (2.739)
Creatinine (mmol/L)	34.466 (32.128)	38.957 (35.689)
FT4 (pmol/L)	3.253 (6.829)	2.736 (6.240)
Glucose (mmol/L)	3.239 (3.236)	3.606 (3.449)
HbA1c (%)	1.495 (2.740)	1.764 (2.941)
Ure (mmol/L)	2.350 (2.862)	2.671 (3.213)
AST (U/L)	15.111 (26.893)	18.649 (50.252)
TSH (mU/L)	0.520 (2.952)	0.405 (1.603)
Calcium (mmol/L)	0.755 (1.097)	0.865 (1.122)
GGT (U/L)	4.304 (20.567)	5.697 (43.397)

**Figure 2:**
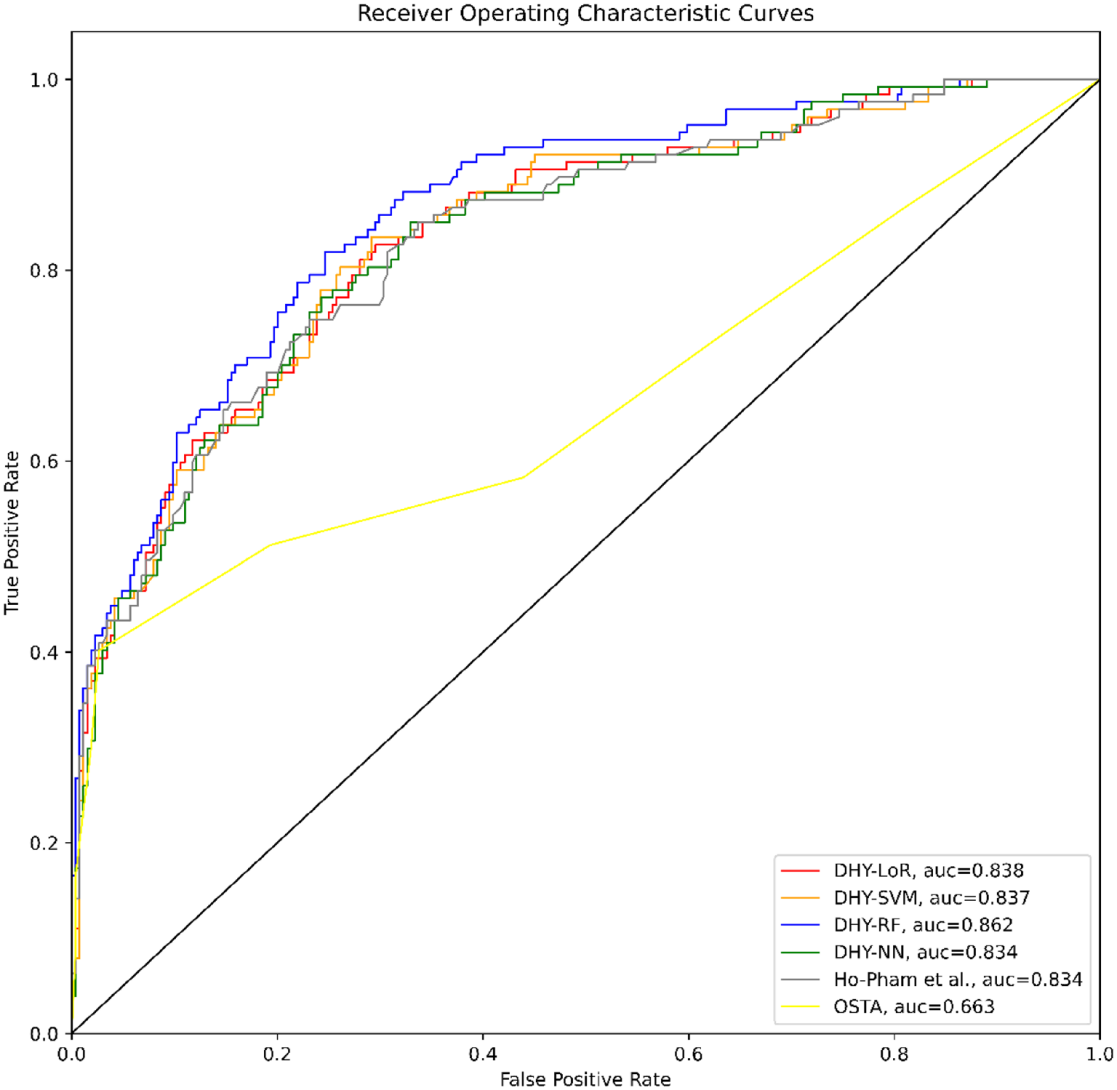
The ROC curves in the first scenarios.

**Figure 3:**
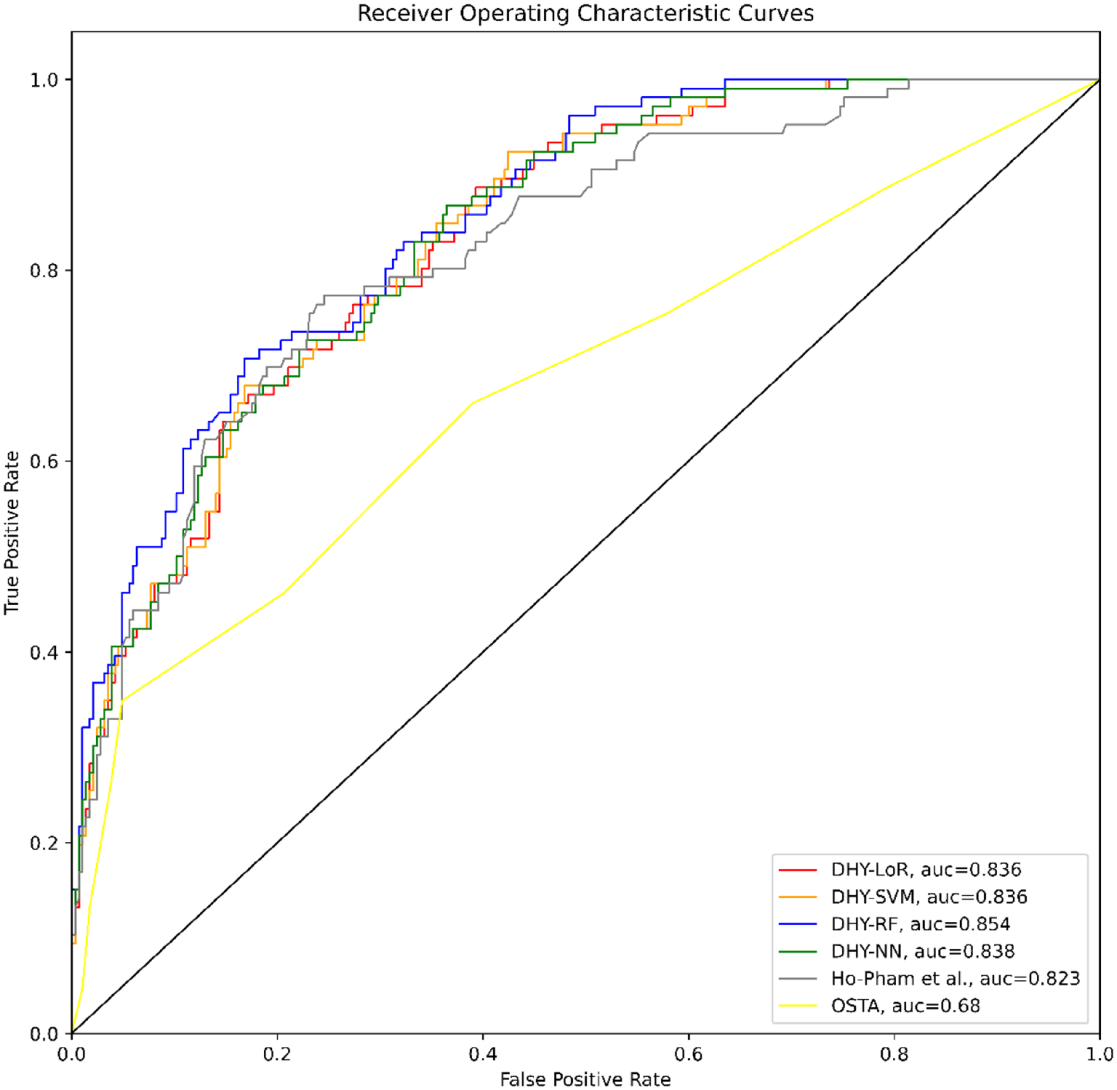
The ROC curves in the second scenario.

Figures 2 and 3 present the results of the first and second scenarios, respectively. While the lines labeled DHY-LoR, DHY-SVM, DHY-RF and DHY-NN represent the ROC curves of four machine learning models—LoR, SVM, RF and NN—applied to the Hanoi Medical University Hospital’s dataset, the grey and yellow lines indicate the LoR method from Ho et al.’s study and the OSTA tools.

Tables 5 and 6 present the performance of included prediction models in the first and second scenarios respectively. The model developed by Ho-Pham et al.^14^ still showed predictive significance using the Hanoi Medical University Hospital dataset (AUROC = 0.823 and AUROC = 0.828). In both scenario, the LoR, SVM, RF, and NN provided clinically significant AUROC values (above 0.8) and outperformed the model of Ho-Pham et al.^14^. The study findings also showed that the OSTA score (AUROC = 0.654 and AUROC = 0.656) had no predictive relevance among the Northern population in Vietnam.

**Table 5:** The performances of the models in the first scenario.

Model	AUROC	Brier score	Precision	Recall	F1 score
LoR	0.832 (0.797–0.857)	0.146	0.545	0.742	0.628
SVM	0.831 (0.797–0.856)	0.147	0.544	0.742	0.628
RF	0.854 (0.825–0.881)	0.137	0.572	0.763	0.653
NN	0.832 (0.791–0.853)	0.147	0.550	0.746	0.633
Ho-Pham et al.	0.823 (0.781–0.851)	0.149	0.489	0.813	0.610
OSTA	0.654 (0.606–0.702)	0.205	0.722	0.353	0.473

**Table 6:** The performances of the models in the second scenario.

Model	AUROC	Brier score	Precision	Recall	F1 score
LoR	0.837 (0.801–0.863)	0.147	0.549	0.738	0.629
SVM	0.836 (0.803–0.862)	0.147	0.552	0.739	0.632
RF	0.845 (0.807–0.875)	0.142	0.577	0.758	0.655
NN	0.837 (0.800–0.862)	0.149	0.550	0.737	0.629
Ho-Pham et al.	0.828 (0.784–0.862)	0.150	0.506	0.826	0.627
OSTA	0.656 (0.621–0.711)	0.206	0.744	0.365	0.489

Besides the AUROC values, several metrics were also calculated to compare the performance of the machine learning models. The precision, recall and F1-score of our models were calculated at the cut-off point determined by Youden’s index. The resultant cut-off value was lower or equal to − 4 for the OSTA model. Overall, our machine learning models performed significantly better than two remaining models on the dataset of the Hanoi Medical University Hospital.

**Figure 4:**
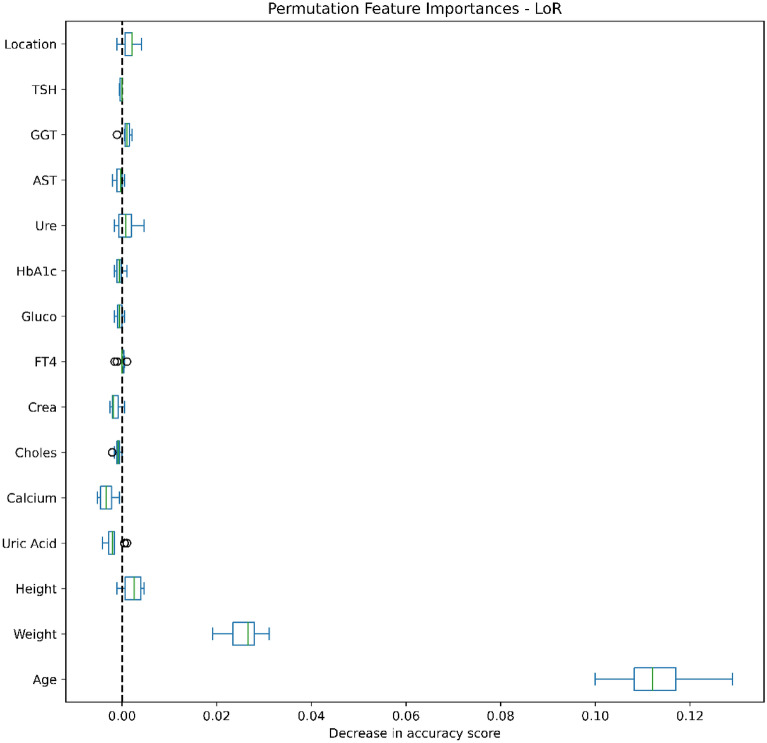
Feature importance for LoR in the first scenario.

**Figure 5:**
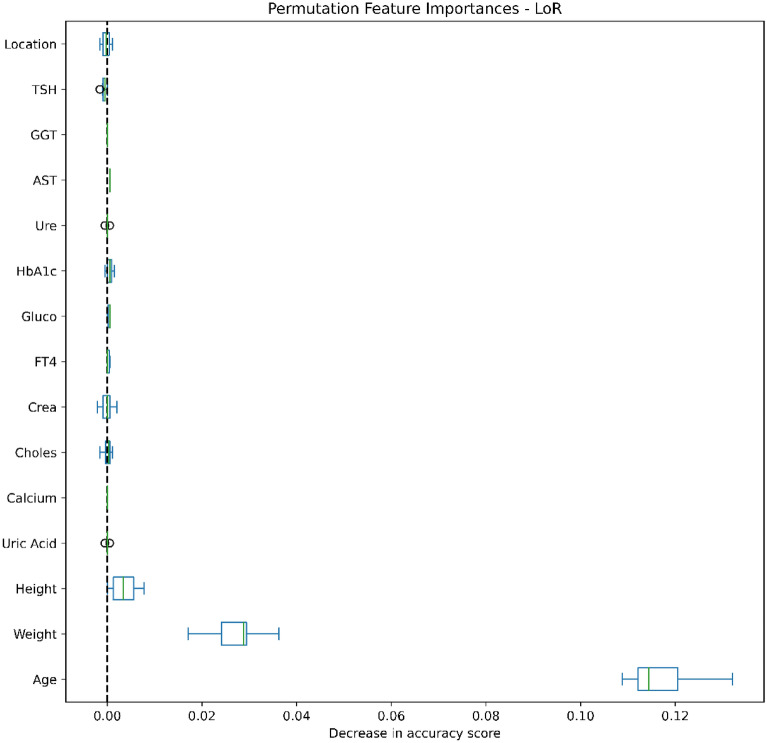
Feature importance for LoR in the second scenario.

**Figure 6:**
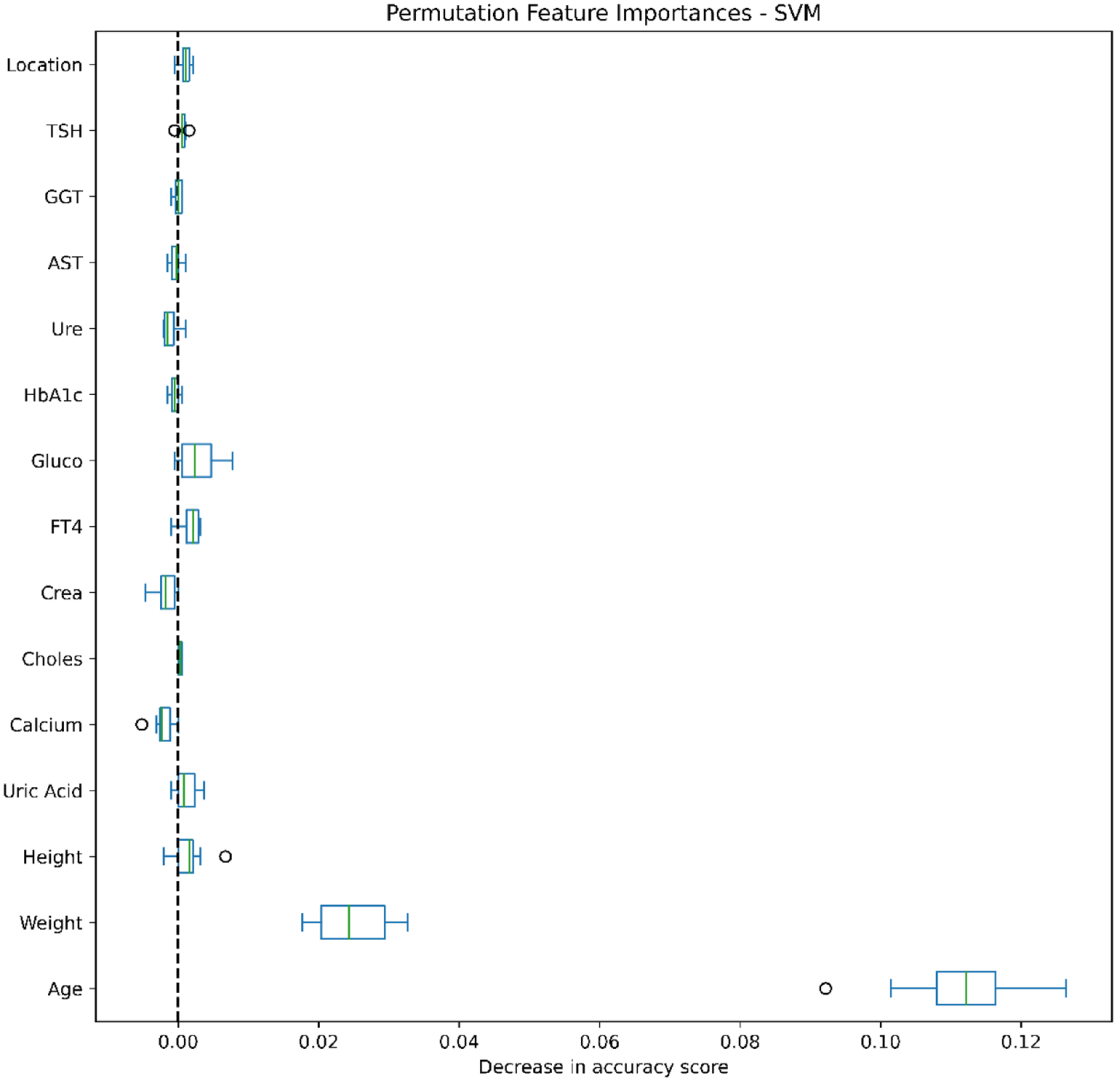
Feature importance for SVM in the first scenario.

**Figure 7:**
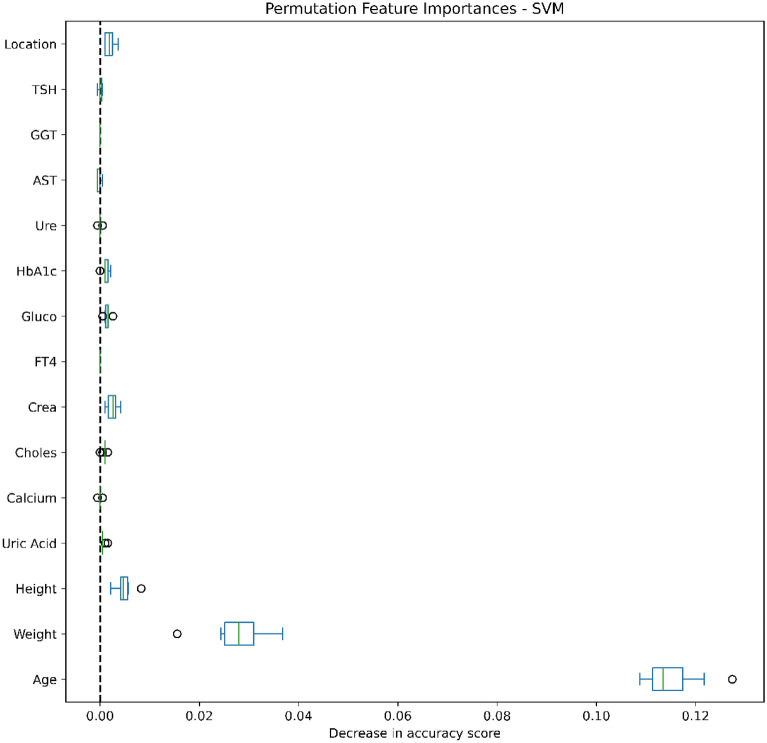
Feature importance for SVM in the second scenario.

**Figure 8:**
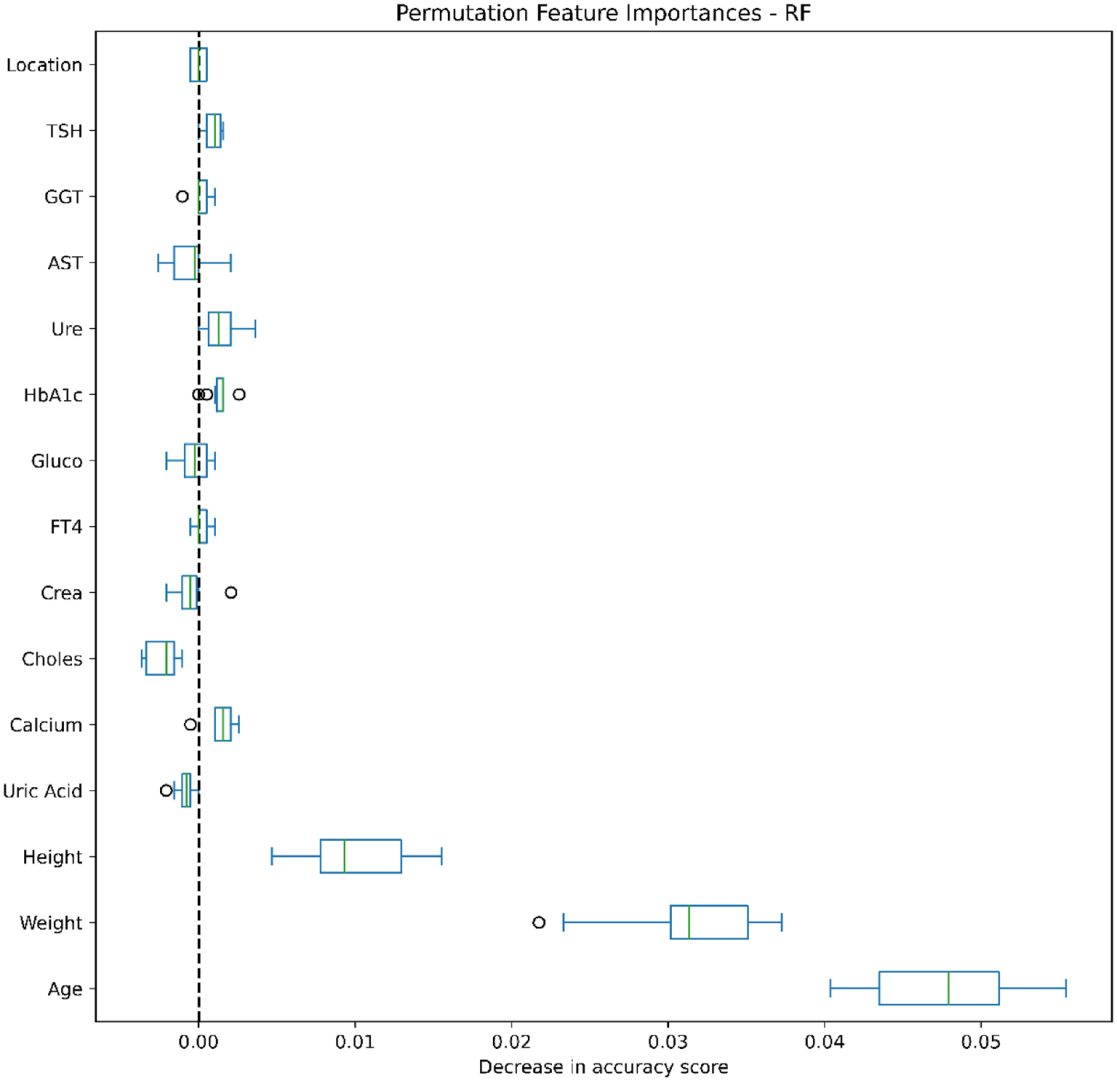
Feature importance for RF in the first scenario.

**Figure 9:**
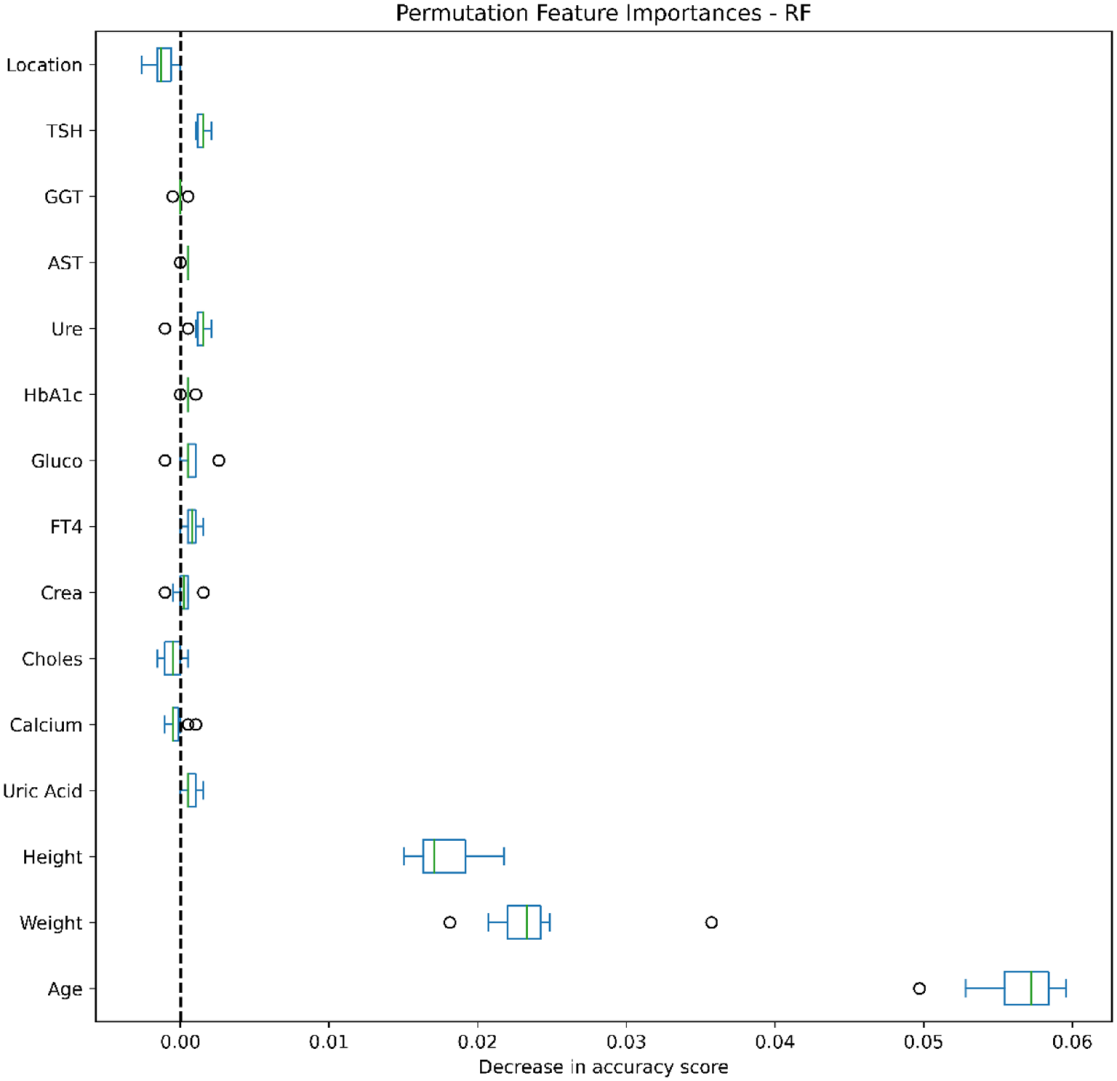
Feature importance for RF in the second scenario.

**Figure 10:**
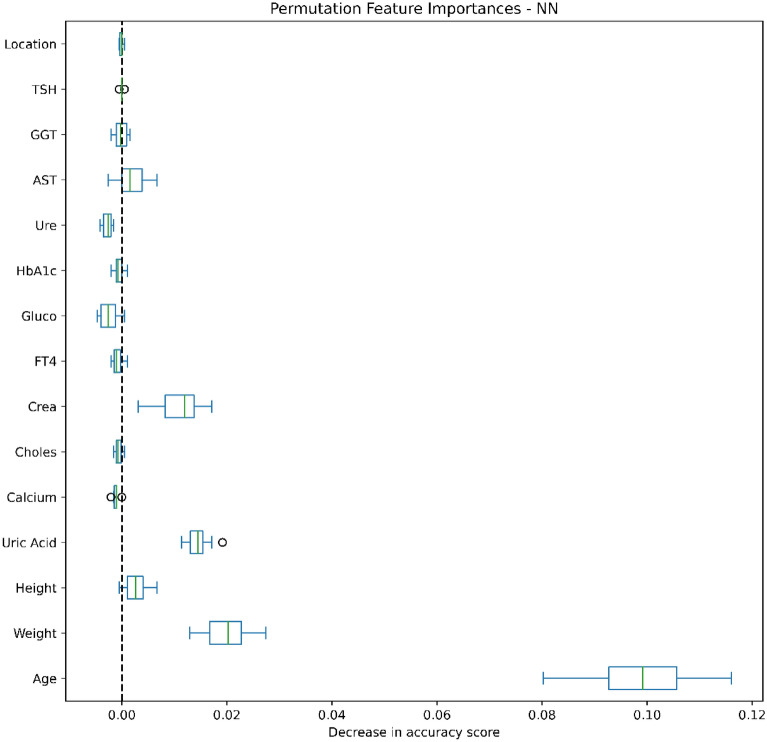
Feature importance for NN in the first scenario.

**Figure 11:**
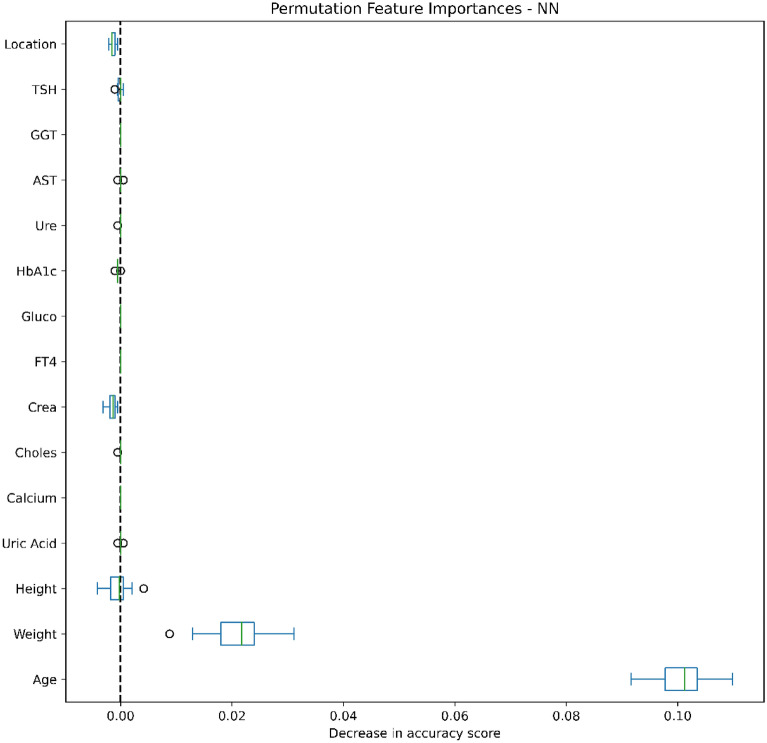
Feature importance for NN in the second scenario.

Figures 4, 5, 6, 7, 8, 9, 10 and 11 present the significance of the various features in the two scenarios. The figures illustrate that the most important predictors of osteoporosis were age and physical condition (height, weight). Although other features, including blood tests and geographical factor, did have an impact on the predicted results, it was not significant in either scenario.

Tables 7 and 8 present the relationship among random features and their outcomes in the first and second scenarios respectively. In the first scenario, when the initial blood test results were taken into account, the only insignificant factor was the participants’ geographical location, and the remaining features were significant in the prediction. In the second scenario, abnormal thresholds of uric acid, cholesterol, creatinine, FT4, HbA1c, AST, GGT, TSH, and geographical location did not affect the results of the model. This can be explained by the very low percentage of patients with abnormalities laboratory results (as mentioned in the previous section) and the small difference in the prevalence of osteoporosis among urban and rural people.

**Table 7:** The relationship among various features and the outcome in the first scenario.

Features in the first scenario	*P*-value
Age	0.00000
Weight	0.00000
Height	0.00000
Uric acid	0.00000
Calcium (CalciTP)	0.01294
Cholesterol (Choles)	0.02913
Creatinine (Crea)	0.00000
FT4	0.00000
Glucose (Gluco)	0.00006
HbA1c	0.00002
Ure	0.00004
AST	0.00000
GGT	0.00000
TSH	0.00090
Location	0.35881

**Table 8:** The relationship among various features in the second scenario.

Features in the second scenario	P-value
Age	0.00000
Weight	0.00000
Height	0.00000
Acid uric	0.08805
Calcium (CalciTP)	0.00266
Cholesterol (Choles)	0.72582
Creatinine (Crea)	0.16660
FT4	0.32249
Glucose (Gluco)	0.04212
HbA1c	0.39439
Ure	0.00004
AST	0.23337
GGT	0.12823
TSH	0.10570
Location	0.35881

## Discussion

Studies have demonstrated the utility of machine learning methods to automatically interpret BMD datasets. While osteoporosis significantly contributes to the health-related burdens in Vietnam, machine learning might provide solutions to the challenge of widening the range of osteoporosis screening. We developed and evaluated four different machine learning tools to detect the absolute risk of osteoporosis in Vietnamese women over age 50. In addition, we evaluated the performance of other published tools that were developed for use in Vietnamese and Asian women as well as analyzed the potential contribution to osteoporosis prediction of different predictors.

We built four machine learning prediction tools that demonstrated promising performance across two scenarios. The AUROC of the four tools indicated a good ability to predict the risk of osteoporosis, with all values reaching above 0.81. The findings of our study in both scenarios were higher than the ones recorded in the study by Erjiang et al. that applied DXA as a testing reference^32^. The NN model was the most accurate prediction tool in both the first scenario of this study and the one by Erjiang et al.^32^. Compared to the study by Ulivieri et al.^40^, the NN model in our study performed better. The ANN in Ulivieri’s study showed good discrimination, with an AUC of 0.8 by external validation^40^. Machine learning approaches were also applied to develop geological prediction models which provided the triage solutions to predict an occurance of rockburst during underground rock excavations^41,42^. We also figured out that the averaged AUROC values of the four tools in our study were higher than ones of FRAX models which were calculated at 0.796 and 0.768 respectively^43^. The FRAX models were evaluated by Fan et al. to predict postmenopausal osteoporosis in 2020^43^.

Similarly to our methods, the indices (precision, recall, F1-core) were estimated to evaluate the performance of machine learning algorithms^41,42^. Ullah et al. developed Extreme Gradient Boosting (XGBoost) model with support from K-means clustering and an enhanced stochastic neighbour embedding (SNE) Based t-SNE algorithm to predict the risk of rockburst^42^. The XGBoost was then evaluated by the estimation of precision, recall and F1-score^42^. In addition, Wojtecki et al. applied 16 machine learning models to forecast the risk of rockburst caused by tremors^41^. The recall, precision, and F1-score were also applied to assess the accuracy of included models^41^.

We included the OSTA and prediction model of Ho-Pham et al.^14^ in our study and conducted the external validation with the aim of validating the models in a new population. The predictive values of OSTA in our study were drastically lower than the figures of Bui et al. for all anatomical sites^3^ and Chen et al. at femoral neck^44^. While the minimum AUC values estimated in the latter studies were 0.709 and 0.830 for OSTA respectively, our findings showed a value of only 0.6427 and 0.6814 in two scenarios^3,44^. The prediction model developed by Ho-Pham et al. proved its good discrimination ability through external validation by our study, with AUC values above 0.8 in both two scenarios^14^. However, the model of Ho-Pham et al. presented the higher estimated Brier scores in our external validation than the one calculated in the internal validation^14^ which indicated the predictive accuracy of the model decreased during our external validation. The difference of estimated Brier scores between two validations might be due to the difference of fixed threshold. The values were at 0.195 and 0.04 for the internal validation conducted by Ho-Pham et al.^14^ while the cut-off used in our study was determined by Youden’s index. Findings by both internal^14^ and external validation suggest promising applications of the tool if applied for community-based screening campaigns.

We also applied Permutation Feature Importance using the included machine learning models in both scenarios to examine the findings of both Yang et al. and Jamal et al., which described potential predictors of osteoporosis prediction in the Vietnamese population^34,35^. Our findings proved that age, weight and height strongly contributed to the risk of osteoporosis, while other predictors were less influential. Our conclusions were further supported by the findings of Ou Yang et al., which found that measured values of age, height and weight in a group with decreased BMD were significantly higher than in a group with normal BMD^34^. However, our findings contrasted with those of Jamal et al., which showed no significant correlations between abnormal test results and risk of osteoporosis^35^. This can be explained by the very low percentage of patients with abnormal laboratory tests and the small difference in the prevalence of osteoporosis in urban and rural people.

There is no disagreement about the exceptional utility of DXA to detect the risk of osteoporosis in facility-based settings. However, there are existing barriers that prevent DXA from being widely implemented in smaller centers. The use of DXA requires significant operation costs, which might result in financial burdens at the national level. To interpret DXA outputs requires highly skilled practitioners who frequently work at tertiary hospitals rather than primary care centers. Moreover, due to its limited mobility, DXA is not suitable for mobile screening events that can promote access to care for people living in remote areas. However, prediction models possess the ability to overcome these challenges through operative advantages. Prediction models could play a role as primary screening tools in the community, in which high-risk subjects could be identified and referred for DXA screening at tertiary hospitals. The smaller number of at-risk patients undergoing DXA scans will reduce the medical cost for both individuals and the government. Moreover, the junior practitioners can be rapidly trained to apply the models during their clinical routine practice at primary care centers. In light of their high mobility, the integration of predictive models will enhance community-based mobile osteoporosis screening.

However, there are existing challenges to incorporating the machine learning model. Firstly, the machine learning models are prone to be deployed as laptop computers or Cloud-based systems that require an internet connection and certain technical maintenance for regular use. This will create technical equipment issues in the case of the mobile screening events. Secondly, the use of the machine learning models might depend on the acceptance of doctors. The disagreement regarding the clinical conclusions between doctors and machine learning model researchers can limit the application in clinical routine practice. Thirdly, the incurred cost to re-evaluate the findings of from machine learning model studies might need analyzing to understand the realistic benefit compared to the potential savings. Lastly, the display language might be worth considering if the machine learning models are to be applied on populations of ethnic minorities.

There are limitations to be considered in our study. Firstly, the validation was not performed on specific anatomical sites (e.g., femoral and lumbar vertebrae), where the speed of bone degeneration might be different from aging. Secondly, an external validation was not conducted to investigate the performance of machine learning models on other populations. Thirdly, the analysis data was collected at one facility, which can limit extrapolation. Lastly, the analysis was not conducted on a subpopulation with comorbidities, which can increase the risk of osteoporosis.

## Conclusion

The dramatic operating cost and the requirement of highly-skilled practitioners have been limited the widely integration of DXA as well as the access-to-care at community level. Machine learning methods have demonstrated its potential to support practitioners in screening for osteoporosis risk in communities. Although there were limitations of validation method and extrapolation, our findings proved the outstanding performance of four machine learning tools to predict osteoporosis among women over age 50. The findings of this study also introduced the decision support techniques which might contribute as primary indicator for community-based osteoporosis screening. To overcome the operating challenges, the tools might be developed as portable computer-assisted devices which might work more flexibly and economically than DXA machines. In addition to the accuracy, there are positive benefits for both in terms of cost-effectiveness and preventive strategies for the policy makers to consider. Additional research should be conducted to further investigate the performance of machine learning models on other specific populations.

## Data Availability

The raw data supporting the conclusions of this study are available upon the request for independent result evaluations from interested parties. The datasets used and/or analyzed during the current study available from the corresponding author on reasonable request.
